# The significance of green exercise for the health and wellbeing of Italian immigrants in Norway: a mixed-methods study

**DOI:** 10.1186/s12889-023-16375-3

**Published:** 2023-08-09

**Authors:** Giovanna Calogiuri, Evi Petersen, Alessio Rossi, Laura Terragni

**Affiliations:** 1 Department of Nursing and Health Sciences, Faculty of Health and Social Sciences, University of South-Eastern Norway, Drammen, Norway; 2 Department of Public Health and Sport Sciences, Faculty of Health and Social Sciences, Inland Norway University of Applied Sciences, Elverum, Norway; 3 Department of Sports, Physical Education and Outdoor Life, Faculty of Humanities, Sports and Educational Science, University of South-Eastern Norway, Bø i Telemark, Norway; 4 Department of Early Childhood Education, Faculty of Education and International Studies, Oslo Metropolitan University, Oslo, Norway; 5 Department of Computer Science, University of Pisa, Pisa, Italy; 6 Department of Nursing and Health Promotion, Faculty of Health Sciences, Oslo Metropolitan University, Oslo, Norway

**Keywords:** Outdoor recreation, Outdoor leisure, Nature exposure, Quality of life, Immigration health, Acculturation, Italian immigrants

## Abstract

**Background:**

Green exercise (physical activity in presence of nature) has beneficial effects for health and wellbeing. Green exercise is a popular form of recreation in the Nordic countries, but participation is lower among the immigrant population from non-Western countries. However, no attention has been given to immigrants from the European Economic Area regarding this topic. Given the cultural and structural differences that surround green exercise in Italy and Norway, the case of the Italian immigrants in Norway is of interest to enrich our understanding of green exercise and its significance for health and wellbeing among immigrants in the Nordic countries.

**Methods:**

This convergent mixed methods study investigated the pathways that link green exercise to health and wellbeing among Italian immigrants in Norway. Quantitative data were collected through an online survey (n = 321), which was oversampled to better reflect the sociodemographic profile of the reference population. Logistic regression was used to model the association of green exercise with self-rated health (SRH) or satisfaction with life (SWL) before and after controlling for selected confounders (age, gender, educational level, language proficiency, social support, and childhood experiences with green exercise). Qualitative data were collected through semi-structured in-depth interviews (n = 14) and analysed thematically. Merging of the two strands was done using a simultaneous bidirectional approach.

**Results:**

The logistic regression found a significant bivariate association of green exercise with both SRH and SWL, though the association remained significant only for SWL after controlling for confounders. From the thematic analysis, three themes were identified: Green exercise opportunities contributing to overall satisfaction, Closeness to nature, and Embracing a new lifestyle. The integrated findings indicate that green exercise supported the immigrants’ wellbeing, especially by providing stress relief, though socioeconomic status and acculturation may have a major impact on general health. Familiarity, appreciation of nature benefits, social support, and acculturation were identified as facilitating factors.

**Conclusions:**

This study provides novel insights into how green exercise supports health, wellbeing, and inclusion among immigrants to the Nordic countries and emphasizes the importance of developing culturally adapted strategies to enhance this health-promoting activity among immigrant populations.

## Introduction

### The health and wellbeing benefits of green exercise

In the past three decades, studies have consistently demonstrated that human-nature interactions provide a broad range of health and wellbeing benefits. Different pathways have been proposed to link nature contact to people’s physical and psychological health, involving air quality/heat modulation, physical activity, social cohesion, and stress reduction [1, 2]. A 2016 review by the World Health Organization demonstrated that natural environments contribute to reducing morbidity and mortality among urban residents through these pathways [3]. In particular, through intertwining and synergic benefits of nature contact and physical activity, green exercise (e.g., visiting naturalistic locations, engaging in outdoor recreation, or exercising or walking in natural environments) has been shown to support health and wellbeing by eliciting desirable psychological, physiological, and social effects [4]. In particular, green exercise is associated with short-term benefits such as improved psychological states [5, 6], reduced psychophysiological stress [5, 7], and facilitation of social interactions [8]. Moreover, when performed regularly, green exercise can elicit long-lasting benefits such as higher levels of self-rated health [9, 10] and satisfaction with life [9, 11], as well as strengthened social support [12, 13]. Different review studies corroborated the added health and wellbeing benefits of green exercise compared to physical activity indoors or in urban settings, though they also highlighted limitation within this research field, including among others the fact that the large majority of studies focus on Western populations [14–16]. On the other hand, evidence exists indicating that the health and wellbeing benefits of nature contact and green exercise can apply to people from various cultures and ethnicities, providing a protective factor for immigrant populations [17]. In light of this, in recent years, the concept of “nature-based integration” has been proposed, especially across Nordic countries, as an efficient and cost-effective way for better integration of immigrants [18]. The research on the health and wellbeing benefits of green exercise among immigrant population in the Nordic context, however, is still in its infancy. In particular, there is a need to understand the way in which immigrants from the European Economic Area (EEA) experience green exercise, and how this contributes to their health and wellbeing.

### Green exercise in the norwegian context

In the Nordic countries, green exercise is culturally valued and embedded within various layers of the societal system, including the educational and sport-related arenas [19]. Particularly in Norway, there is a vibrant tradition for green exercise, known as “friluftsliv”. This unique cultural phenomenon, prevalent in all Scandinavian countries, has roots in a historical lifestyle and means of transportation. It has evolved into a traditional leisure practice where being in and moving through nature using simple means is a core motivational element [20]. Today, green exercise in Norway is largely popular, also supported by the fact that 61% of the population has access to safe recreational areas and natural environment [21]. Moreover, green exercise practice is reinforced by policies granting access to natural environments and promoting the practice within compulsory school and health institutions [22]. A 2016 study showed that 61% of Norwegians engaged in green exercise during a regular week [23]. Walks and hikes in the woods or mountains are especially popular, with water-related activities (e.g., walking by coasts or kayaking) and winter activities (e.g., downhill or cross-country skiing) being popular seasonal activities [24]. In an immigration context, studies show that immigrants residing in Norway generally appreciate and participate in green exercise [25–27]. For instance, a qualitative study on female immigrants from various backgrounds residing in Norway revealed a complex use and perception of natural environments for recreation, physical activity, and social purposes, generally supporting the women’s mental health [26]. On the other hand, immigrants were found to often experience barriers to the practice of green exercise, including lack of knowledge on where and how to practice it, challenges in understanding information on advertised opportunities, lack of time, and fatigue [27]. A case study also found that, compared to the non-immigrant population, immigrants living within Oslo metropolitan area had less daily access to areas that can support green exercise [28]. The Norwegian Government formally acknowledged the vital role of green exercise among immigrants and prompts its promotion within its official integration strategy framework [23].

### Green exercise and acculturation

As green exercise is an important part of the Norwegian culture and way of life, the practice of this activity among immigrants can be seen under the prism of acculturation. A widely used definition of acculturation refers to “the process of cultural and psychological change that takes place as a result of contact between cultural groups and their individual members” [29]. Acculturation can take multiple and dynamic paths [30]. Immigrants may adopt aspects of the culture of the host country in some spheres of their lives, but not in others, or adapt aspects of the host couture to their own. Acculturation’s path may be influenced by the specific reasons for migration, how immigrant groups are received in the host country, and personal resources. Differences among groups of immigrants with different backgrounds have been highlighted [27, 31] and, with the few existing studies mainly focusing on immigrants from non-Western countries, a knowledge gap remains with respect to the practice of green exercise and its significance to health and wellbeing among intra-EEA immigrants.

### EEA migration in the nordic countries: the case of italian immigrants in Norway

While immigrants from Asia, Africa or Latin America account altogether for half of the immigrant population in Nordic countries, the remaining part is predominantly from countries within the EEA [32]. Among these, in 2023, Norway counted 6,112 immigrants of Italian origins (3,724 males and 2,388 females), of which 5,700 were first-generation immigrants [33]. Although a relatively small group, it should be considered that Italian migration to Norway has been steadily increasing since the EEA agreement in 1994 and has tripled since the economic crisis in 2008 [33]. A peculiarity of this group is that, compared to other immigrant groups, most of Italian immigrants in Norway have a higher educational level and are employed in high-income occupations. For instance, in 2019, it was estimated that 61% of Italian immigrants in Norway had a university degree, with 17% possessing a Doctoral degree [34] –by comparison, the proportion of individuals with a university degree in Italy and Norway in the same year was 20% and 33%, respectively [35, 36]. However, many Italian immigrants in Norway work in less stable and more menial jobs [37]. Although among the Italian immigrants in Norway there is a considerably larger prevalence of men than women, the proportion of female immigrants, including women moving alone for occupational or study purposes, has been increasing in the past decade [37]. These trends are largely in line with immigration trends from other Southern-European countries, such as Spain and Greece [32].

### Health and wellbeing among italian immigrants in Norway

Immigrants are often at higher risk for lower quality of life than the host country’s general population [38]. In the Norwegian context, a 2016 national survey among immigrants found that, compared to the non-immigrant population, immigrants often reported more frequent mental health challenges, lower self-rated health, and lower life satisfaction [39, 40]. Such health challenges have been associated with poor language proficiency, unfamiliarity with the health system, or limited social networks [41–43]. Moreover, differently than in the non-immigrant population, for which life satisfaction was primarily predicted by sociodemographic factors (primarily educational level), among immigrants life satisfaction was primarily predicted by immigration-related factors, such as having a partner living abroad and experiencing discrimination [40, 44]. Relatively to the Italian immigrants in Norway, a recent study found equivalent levels of self-rated health as compared to both the general Norwegian and Italian populations [34]. However, considering the higher socio-economic status of this group [45, 46], which is generally associated with *better* health, this finding indicate the presence of some health-related challenges. Indeed, Italian immigrants in Norway were found to experience lower levels of health literacy [47] as well as challenges in navigating the Norwegian social norms and systems, leading to barriers in using the health system and establishing meaningful social relationships [46]. On the other hand, many (especially among the women) reported to have increased their physical activity levels after moving to Norway [25]. In particular, 64% of the Italian immigrants in Norway practiced some green exercise within a regular week, a prevalence that is equivalent to the Norwegians’ [25]. This contrasts with the much lower levels of green exercise participation among Italians living in Italy, where only 25% reported to perform, regularly or occasionally, green exercise in urban settings (e.g., parks, but also city streets), while 31% do it in natural environments outside the city (e.g., mountains, sea- or lakeshores, forests). These patterns are likely related to the poorer availability of green exercise opportunities that characterize Italy, which is among the European countries with the lowest population-weighted surface area of natural environments that can be reached within 10 min of walking [48]. Nevertheless, this corroborates the assumption that the Italian immigrants tend to change their green exercise habits after resettling in Norway, which may contribute buffering the health and wellbeing challenges that they face.

### The present study

Given the cultural and structural differences that characterize Italy and Norway concerning the practice of and opportunities for green exercise, as well as the increased green exercise participation observed among the Italian immigrants in Norway, the case of this particular migration group is of interest to gain a better understanding of green exercise and its health and wellbeing benefits in the context of migration within (though not limited to) the Nordic countries. Furthermore, representing a case of intra-EEA migration, the focus on Italian immigrants in Norway can extend the knowledge of this under-researched group. Hence, the overarching purpose of this study was to investigate the extent to which green exercise may support health and wellbeing among Italian immigrants to Norway, as well as to explore factors that may influence this relationship. Specifically, the present study used a mixed-methods approach to provide both a broad overview of the phenomenon with generalizable findings and an in-depth understanding of the Italian immigrants’ experiences and beliefs.

## Materials and methods

### Overall design

The present study is part of the larger project “Mens Sana in Corpore Sano”, which investigated health and health-related behaviours of Italian immigrants in Norway [49]. The data collection was conducted between March and May 2019 (i.e., before the Covid-19 lockdown). The study adopted a convergent mixed method design [50, 51]. To facilitate the integration process, the survey and the interview guide were purposefully developed based on the same topics (e.g., participation in and experiences with green exercise in Italy and in Norway). The quantitative and qualitative strands were conducted and analysed in parallel, and the findings of each strand presented separately [50]. The merging was done with a simultaneous bidirectional approach, using a matrix to compare the outcomes of the statistical analysis with the qualitative quotes and themes extracted from interviews [51]. Equal relevance was given to both strands, focussing on whether there was consistency between them and how the outcomes of each strand may expand the other. The initial merging frame was outlined by one author (GC) and further developed in collaboration with two other authors (LT and EP). The final interpretation and reporting of the integrated findings were done by discussing both qualitative and quantitative findings together on a theme-by-theme basis [50].

### Quantitative strand

#### Data and participants

The survey was conducted among adult (18 years or older) Italian immigrants residing in Norway, who spoke Italian and spent most of their childhood (up to age 16 years) in Italy. Since an updated contact list of all the Italians living in Norway was not available, the survey was distributed through different channels, including invitations through the mail-list of COMITES Oslo (an elected representative body of the Italian community) and announcements on the Italian Embassy’s newsletter, and different online groups for Italians living in Norway. Compliance with the inclusion criteria was assessed through control questions in the survey. A total of 330 people responded to the survey, of which 321 met all the above criteria. A comparison of sociodemographic variables of the sample with figures provided by Statistics Norway and the Registry of Italian Citizens Residing Abroad revealed that the sample had a larger proportion of women, mid-aged individuals, people with a higher educational level, and people living in the region of Oslo-Akershus (i.e., the most populated region of Norway, containing Norway’s capital Oslo). To enhance the sample’s representativeness, the original dataset was oversampled using the ADASYN method [52] based on the expected distribution of age and education level (final n = 531), which also resulted in an acceptable adjustment of gender and place of residence. Details about the oversampling process and sample’s characteristics are reported in a previous publication [25].

#### Instruments and Study variables

The questionnaire used in the Mens Sana in Corpore Sano study included items retrieved from surveys previously conducted by professional statistical agencies in the Norwegian population. All items were translated to Italian by native speakers with expertise in health surveys. Besides contributing to higher validity of the measurements, this also allowed for comparisons with other immigrant groups or the general Norwegian population (see previous publications [25, 34, 47]). More specifically, the items included in in this particular study were retrieved from the following surveys:

I. *Levekårsundersøkelsen blant personer med innvandrerbakgrunn 2016* (“Survey on living conditions among people with immigration background 2016”) [39]. This survey was designed and distributed by Statistics Norway to investigate the health and living conditions of immigrants from non-western countries. The survey contains many of the items included in the *Levekårundesøkelsen om helse* (“Survey on living conditions”), a survey routinely conducted by Statistics Norway in the Norwegian general population [53];
II. FRIFOs aktivitetskartlegging 2012 (Physical activity survey for FRIFO 2012), a survey on physical activity habits and related beliefs and motivational factors in the general Norwegian population, which was initiated by The Norwegian Outdoor Council and designed and conducted by Ipsos MMI [23].

For the present study, the following variables were used:

**Outcome variables.** The outcome variables were health and wellbeing, operationalized as Self-rated health (SRH) and Satisfaction with life (SWL), respectively. SRH was assessed through a single-item inquiring “In general, how would you rate your health?” which was rated on a 5-points liker scale (1 = Very bad, 2 = Bad, 3 = Neither bad nor good, 4 = Good, 5 = Very good). Similar instruments are commonly used in population studies as a subjective and comprehensive indicator of health, and studies have shown their validity against objective assessments of mortality [54, 55]. SWL is an overarching construct that captures the affective feelings and cognitive judgments people have about the quality of their lives, which has received growing scientific attention as an indicator of wellbeing as well as immigration’s overall success [40, 56]. In this study, SWL was assessed through an item inquiring “Overall, how satisfied are you with your life at the moment?” which was rated on an 11-points visual scale (0 = Absolutely unsatisfied; 10 = Absolutely satisfied). Both instruments were previously used in a Norwegian survey on living conditions among immigrants [39, 40] as well as in other studies investigating the association of green exercise with health and wellbeing [9, 10].

**Primary predictor.** The main predictor in this study was Green exercise, a measurement that grossly quantified the frequency with which the Italian immigrants participated in this activity, which was assessed through three items (Table 1). Two items were designed to capture the extent to which the participants performed green exercise activities that are common in the Norwegian context and more generic experiences in nature. The third item provided a gross indication of the respondents’ amount spent in green exercise relative to their overall weekly physical activity. A caption stated “Consider the overall time you spend doing physical activity during a regular week. How much of this time do you spend in each of the following activities:” A list of activities was then presented, including “Walking or exercising in parks, green spaces, or other natural environments”. Each item was rated on a 4-points scale (Table 1). The internal consistency of the measure was adequate (Cronbach’s α = 0.70).

**Table 1:** Individual items for the compound predictors included in the logistic regression model

Individual item	n (%)	
**Green exercise (α = 0.70)**		
“I often practice activities in nature such as walking in the woods or countryside, hiking in the maintains, activities on/by the sea, etc.”		
- It does not fit me	63 (11.86)	
- It fits me a little	228 (42.94)	
- It fits rather well	168 (31.64)	
- It fits me very well	72 (13.56)	
“I often find myself in the quietness of nature”		
- It does not fit me	87 (16.38)	
- It fits me a little	220 (41.43)	
- It fits rather well	160 (30.13)	
- It fits me very well	64 (12.05)	
“Walking or exercising in parks, green spaces or other natural environments” [proportion of overall time spent in physical activity during a regular week]		
- None	190 (35.78)	
- Less than half	182 (34.27)	
- About half	85 (16.01)	
- More than half	74 (13.94)	
**Childhood experiences with green exercise (α = 0.78)**		
[As a child, up to 16 years] “I often practiced activities in nature such as walking in the woods or countryside, hiking in the maintains, activities on/by the sea, etc.”		
- It does not fit me	111 (20.90)	
- It fits me a little	192 (36.16)	
- It fits rather well	152 (28.63)	
- It fits me very well	76 (14.31)	
[As a child, up to 16 years] “I often found myself in the quietness of nature”		
- It does not fit me	247 (46.60)	
- It fits me a little	163 (30.75)	
- It fits rather well	85 (16.04)	
- It fits me very well	35 (6.60)	
**Social support for green exercise (α = 0.76)**		
“I know many people who practice green exercise”		
- It does not fit me	33 (6.23)	
- It fits me a little	117 (22.08)	
- It fits rather well	253 (47.74)	
- It fits me very well	127 (23.96)	
“I am often invited to participate in green exercise”		
- It does not fit me	191 (36.45)	
- It fits me a little	186 (35.50)	
- It fits rather well	114 (21.76)	
- It fits me very well	33 (6.30)	
“I often practice green exercise with friends”		
- It does not fit me	209 (39.58)	
- It fits me a little	211 (39.96)	
- It fits rather well	85 (16.10)	
- It fits me very well	23 (4.36)	

**Control variables.** Control variables were included in the analyses to assess the extent to which they may influence and explain the relationship of Green exercise with SRH and SWL. These variables, which were identified based on previous scientific literature as well as exploratory analyses, included measurements of green exercise supporting factors (Childhood experiences and Social support), sociodemographic characteristics (Age, Gender, and Educational level), and acculturation (Language proficiency). Childhood experiences and social support are known to influence people’s participation in green exercises [13, 57], as well as its relationship with health and wellbeing [1, 4]. Childhood experiences were assessed through two items, which were similar to those used to assess Green exercise, but specifically referring to the respondents’ childhood (Table 1). Social support was assessed through three items relative to social facilitation for the practice of green exercise (Table 1). The Internal consistency of both measurements was acceptable (Cronbach’s α = 0.78 and 0.76, respectively; Table 1). Individuals’ sociodemographic characteristics are known to influence health and participation in physical activity, including green exercise [34, 47]. In this study, the following sociodemographic characteristics were included as possible confounders: Age (in years), Gender (male or female), and Educational level (Upper-level school or lower, University degree [Bachelor, Master, or equivalent], or Doctoral degree [Ph.D. or equivalent]). In line with previous research, Language proficiency was used as a proxy measure for acculturation [58, 59]. This was assessed through a single-item inquiring “How would you rate your Norwegian-language skills?” The item was rated through a 5-pt Likert scale (1 = Very poor; 2 = Poor; 3 = Neither poor nor good; 4 = Good; 5 = Very good).

### Statistical analysis

In line with previously used approaches [9, 60], SRH was dichotomized as ‘worse SRH’ (response options “Very bad”, “Bad”, and “Neither good nor bad”; n = 164) and ‘better SRH’ (response options “Good” and “Very good”; n = 367), while SWL was dichotomized as ‘lower SWL’ (ratings < 7; n = 192) and ‘higher SWL’ (ratings ≥ 7; n = 339). All variables were explored for distribution, missing data, and outliers. No missing data or extreme outliers were identified. Cronbach’s alpha (α) was calculated to assess the internal consistency of the multi-item variables (Green exercise, Childhood experiences, and Social support). Descriptive statistics were performed and presented as means (*M*) and standard deviation (*SD*) or frequency (*n*) and percentages (*%*). Assumptions for multivariate logistic regression were preliminary assessed. All observations were independent of each other, and the sample size was deemed adequate for a multivariate analysis based on the ‘rule of thumb’ of at least 10–20 observations for each predictor included [61]. Multicollinearity was evaluated through the Variance Inflation Factor and linearity in the logit for the continuous variables was evaluated using the Box-Tidwell test. Additionally, exploratory bivariate regressions were conducted to evaluate the relationship of SRH or SWL with all control variables individually. For each outcome variable (SRH and SWL), two logistic regression models were performed to establish the relationship of Green Exercise with better/worse SRH and higher/lower SWL. Firstly, bivariate logistic regression was performed to establish the un-controlled relationship of Green exercise with each outcome variable. Subsequently, to assess the extent to which other factors influence the relationship of Green Exercise with the outcome variables, a multivariate model was performed including Green Exercise as a predictor whilst controlling for all other variables (Childhood experiences, Social support, Gender, Age, Educational level, and Language proficiency). The explained variance for each logistic regression model was expressed as Negelkerke’s pseudo *R*^*2*^, while the effect size of the association for the individual predictors was expressed as odds ratio (*OR*) and 95% confidence intervals (*95% CI*).

All analyses were performed using IBM SPSS Statistics version 27.0 (Chicago, IL, USA). Significance level was set at *p* < 0.005. The statistical analysis was conducted by one author (GC) and revised by a second author (AS).

### Qualitative strand

#### Participants

Informants were recruited using snowball sampling. Inclusion criteria were the same as for the quantitative strand. The process was initiated through an announcement during a gathering of Scienze senza confini (“Science without borders”), an initiative promoted by COMITES Oslo an that aim at connecting Italian professionals living in Norway. The recruitment process aimed to represent the variation among Italians living in Norway concerning gender, age, years of permanence, and reasons for migrating. The final sample consisted of 14 participants (eight women and six men), half of whom have lived in Norway for more than 10 years, while four had lived in Norway between 5 and 10 years and three for less than 5 years. Four were between 30 and 40 years old, eight between 41 and 50 and the remaining were between 51 and 65 years old. Three of the informants moved to Norway for study reasons, three for family reasons, two to follow their partners, and the remaining for work-related reasons. Most of the participants lived in Oslo.

#### Data collection

In-depth interviews were chosen as this methodology offers the opportunity to explore a phenomenon’s more thoroughly and flexibility to ask relevant follow-up questions [62]. The interview guide focused on the health and life conditions of the Italian immigrants in Norway, and particular attention was given to the Italian immigrants’ attitudes towards and experiences with green exercise. The interview guide was designed by the last author (LT), who has expertise on qualitative research and migration’s health, in dialogue with the first author (GC), who has wide expertise in green exercise as well as barriers to and the salutogenic effects of human-nature interactions. The interview guide was pilot tested on two individuals, to check for fluidity of the interviews and refine questions. No relevant adjustments were made to the interview guide after the pilot testing, hence the two pilot interviews were included in the final sample. All interviews were conducted in Italian (by a research assistant or the last author). To better accommodate the schedule of the informants and reach residents in various parts of the country, most interviews took place via video call, at the informants’ homes, or in public places (e.g., a cafés or park). Each interview lasted 45 to 90 min. One author (LT) transcribed the interview audio files verbatim. Since the Italian community is relatively small, to ensure high privacy standards, only this author had access to the complete interview audio files and transcripts.

#### Data analysis

The interview data were analysed using a thematic analysis approach [63], which is coherent with the mixed-methods research design, as it facilitates communication among researchers who use different research methods [64]. In this study, we have focused on how respondents experienced green exercise and the way it influenced their health and wellbeing. After familiarising with the data, initial codes were generated using a hybrid approach of inductive and deductive coding and theme development [65]. An initial coding frame was developed based on the study’s research questions and the interview guide’s main themes (e.g., experiences with nature). In the next step, inductive codes were generated directly from the text to develop the sub-themes (e.g., closeness to nature) or to identify novel themes (e.g., green exercise Italian style). The final refinement of themes occurred through dialogue among three authors (LT, EP, and GC), alongside reiterative reading and reflections based on the literature.

## Results

### Quantitative findings

Descriptive statistics for all the variables, for the overall sample as well as for the participants with worse/better SRH and lower/higher SWL, are presented in Table 2.

**Table 2:** Descriptive statistics of all study variables

Variable	Overall sample (n = 531)	SRH		SWL	
		Worse (n = 164)	Better (n = 367)	Lower (n = 192)	Higher (n = 339)
Green exercise, **M ± SD**	1.98 ± 0.75	1.78 ± 0.73	2.06 ± 0.75	1.70 ± 0.7	2.13 ± 0.73
Childhood experiences, **M ± SD**	2.10 ± 0.86	2.00 ± 0.84	2.14 ± 0.87	2.11 ± 0.79	2.09 ± 0.9
Social support, **M ± SD**	2.24 ± 0.71	2.04 ± 0.69	2.33 ± 0.71	1.97 ± 0.71	2.40 ± 0.67
Age, **M ± SD**	40.45 ± 10.79	40.80 ± 11.68	40.42 ± 10.38	39.82 ± 11.19	40.94 ± 10.55
Gender, **n (%)**					
Male	321 (64.5)	106 (65%)	215 (59%)	123 (64%)	198 (58%)
Female	210 (39.55)	58 (35%)	152 (41%)	69 (36%)	141 (42%)
Educational level, **n (%)**					
Upper-level school or lower	205 (38.60)	83 (51%)	122 (33%)	106 (55%)	99 (29%)
University degree	234 (44.07)	67 (41%)	167 (46%)	67 (35%)	167 (49%)
Doctoral degree	92 (17.33)	14 (9%)	78 (21%)	19 (10%)	73 (22%)
Language proficiency, **M ± SD**	2.99 ± 1.23	2.7 ± 1.28	3.12 ± 1.19	2.66 ± 1.17	3.18 ± 1.23

*Notes*:

SRH = Self-rated health (Worse levels = Very bad/Bad/Neither bad, nor good; Better levels = Good/Very good); SWL = Satisfaction with life (Lower levels = ratings 0–6; Higher levels = ratings 7–10); University degree = Bachelor’s degree, Master’s degree, or equivalent; Doctoral degree = Ph.D. or equivalent

Table 3 presents the outcomes of the logistic regressions examining the relationship of Green exercise with SRH and SWL, before and after controlling for the confounders. The bivariate logistic regression revealed a statistically significant and positive association of Green exercise with both SRH and SWL, indicating that the participants who practiced green exercise more frequently had a greater likelihood of reporting ‘better SRH’ and ‘higher SWL.’ In the multivariate models, the relationship of Green exercise with SRH was no longer significant, indicating that the SRH’s levels were better explained by the control variables. In particular, the model indicates that the respondents’ Educational level and Language proficiency were significant and independent predictors of SRH, with those having the highest educational degree (Doctorate or equivalent) and those with better proficiency of the Norwegian language being more likely to report better SRH compared to those with a lower educational degree (Upper-level school or lower) and poorer proficiency in the Norwegian language. Differently, Green exercise remained a highly significant predictor of SWL even after controlling for multiple control variables. Alongside Green exercise, Childhood experiences and Social support also were identified as significant predictors of SWL, indicating that these factors play an important and independent role within the pathways that link Green exercise to SWL. Social support showed a positive relationship with SWL, with those perceiving to have a stronger social support for green exercise being more likely to report higher SWL. On the other hand, Childhood experiences showed a negative relationship with SWL, indicating that those who had less frequent green exercise experiences during childhood were more likely to experience higher SWL. Higher Education level and Language proficiency were also significantly and positively associated with higher SWL.

**Table 3:** Bivariate and multivariate logistic regression modelling the relationship of Green exercise with SRH and SWL among Italian immigrants in Norway (n = 531)

Predictor	*SRH*			*SWL*		
	*W*	*OR (95% CI)*	*p*	*W*	*OR (95% CI)*	*p*
***Bivariate model***	*(Negelkerke R*^*2*^ *= 4%)*			*(Negelkerke R*^*2*^ *= 10%)*		
Green exercise	14.55	1.67 (1.28–2.17)	< 0.001	35.51	2.26 (1.73–2.96)	< 0.001
***Multivariate model***	*(Negelkerke R*^*2*^ *= 11%)*			*(Negelkerke R*^*2*^ *= 22%)*		
Green exercise	2.87	1.30 (0.96–1.77)	0.090	14.18	1.82 (1.33–2.48)	< 0.001
Childhood experiences	0.82	1.12 (0.88–1.41)	0.366	4.30	0.78 (0.62–0.99)	0.038
Social support	3.10	1.36 (0.97–1.90)	0.079	7.66	1.63 (1.15–2.30)	0.006
Age (yrs.)	0.43	0.99 (0.98–1.01)	0.513	1.51	1.01 (0.99–1.03)	0.220
Gender						
Man	-	-	-	-	-	-
Woman	0.90	0.81 (0.52–1.25)	0.343	1.99	0.73 (0.47–1.13)	0.158
Educational level:						
Upper-level school or lower	-	-	-	-	-	-
University degree	0.91	1.24 (0.80–1.93)	0.340	8.57	1.95 (1.25–3.04)	0.003
Doctoral degree	8.54	2.74 (1.39–5.40)	0.003	10.66	2.89 (1.53–5.45)	0.001
Language proficiency	6.71	1.25 (1.06–1.48)	0.010	7.76	1.27 (1.07–1.51)	0.005

*Notes*:

SRH = Self-rated health (0 = Worse, 1 = Better); SWL = Satisfaction with life (0 = Lower, 1 = Higher); University degree = Bachelor’s degree, Master’s degree or equivalent; Doctoral degree = Ph.D. or equivalent.

### Qualitative findings

From the qualitative analysis, three themes were identified, each containing two sub-themes: *i*. Green exercise opportunities contributing to overall satisfaction (Appreciation of everyday nature experiences and Green exercise supporting health and wellbeing); *(ii)* Closeness to nature (Feeling surrounded by nature and Tamed nature vs. Wilderness); and *(iii)* Embracing a new lifestyle (Changing mind-set and Outdoors activities ‘Italian Style’).

### Green exercise opportunities contributing to overall satisfaction

**Appreciation of everyday nature experiences.** In several interviews, it became visible that Italians living in Norway experience nature in their everyday life, which was generally appreciated. In contrast to their past life in Italy, views of nature from home or other everyday locations became a new routine in Norway. The voice of a participant depicts well how such everyday nature experiences, as opposed to more “extraordinary” nature experiences, are coloured by positive evaluations:

*“Let’s say that I haven’t visited much of Norway, I would like to live it a bit more because I really like it. I cannot put myself in extreme situations, but for a person coming from [a big city in Italy] the idea of having a deer coming to eat in my garden it’s a unique feeling.” (Woman, 40 years)*.

**Green exercise supporting health and wellbeing**. The Italian immigrants reported to perceive the presence of nature in their everyday life as a factor contributing to their overall wellbeing, mainly by providing opportunities to relax and get away from daily hassles. Further, relaxing was recurrently reported as an important motive for seeking nature contact by engaging in green exercise.

*“For me, the best is finding a nice place outside, escaping the rhythm of the city […] it is priceless. It contributes very much to feeling well. The sight of this beautiful nature relaxes me a lot. It helps my wellbeing a lot.” (Woman, 50 years)*.

As an informant observed, green exercise is perceived as embedded within general strategies for promoting health in Norway.

*“In Italy, if you have a health problem, they put you on sick leave for a week, but here they tell you: go for a walk in the forest!” (Woman, 48 years)*.

### Closeness to nature

**Feeling surrounded by nature.** Another recurrent theme in the qualitative interviews was the perceived accessibility of nature in Norway compared to Italy. Accessibility to nature was described not just as shorter distances to environments where one can practice green exercise but also through the felt opportunity of feeling close to nature:

*“Nature, I used it also earlier [in Italy], this must be said, [here] it’s not so much that we use it, we are inside it. The houses, even though you are in the city, you still are in close contact with nature.” (Man, 58 years)*.

*“Here I have it so close that is impossible not to perceive it, […] you basically have it inside your home. You step outside and you are in the middle of nature, you take the subway and are in Sognsvann [the entrance to a popular hiking area], you take the ferry and are on the islands.” (Woman, 40 years)*.

**Tamed nature vs. Wilderness.** In an interview, an informant described how the natural landscape in Italy is dominated by agricultural activities (e.g., vineyards or cultivated fields). In Norway, nature is perceived as more uncontaminated or wild, even in the vicinity of cities. These landscapes appear to have the potential to change the experience of nature when engaging in green exercise.

*“Let’s say that here there is a more direct contact with the wilderness. I used to live in Tuscany and, even if I did bike rides, I did it among cultivated lands, environments more influenced by human activity, and instead here, with short distance to the forest and more uncontaminated and untamed nature, this changed. And I tend to enjoy it more.” (Woman, 35 years)*.

#### Embracing a new lifestyle

**Changing mind-set.** In public discourses, Norwegian nature is primarily interpreted as a safe place, not only in practical terms (i.e., lack of dangers) but also culturally: spending time in nature is part of a core of shared norms in the Norwegian society. The Italian immigrants seem to have partially absorbed this culture, learning new forms of green exercise that are iconic in Norwegian society (such as cross-country skiing) or spending their spare time outdoors with friends or family. One informant talked about “changing their mindset” after moving to Norway and seeing new opportunities. Activities like downhill skiing, which in Italy were seen as activities restricted to holiday periods or weekends, became part of the everyday exercise routines in Norway.

*“I had to change my way of thinking. Hence, for the second year in a raw I bought the season-pass to go skiing, because I do enjoy down-hill skiing […]. This year I even went by myself, because my children do other activities, so yes, my winter activity is down-hill skiing.” (Woman, 50 years)*.

Changing mindset regarding green exercise practice also was mentioned when informants talked about getting accustomed to being outdoors, even in more adverse weather conditions than those they used to tolerate in Italy.

*“Before, in Italy, if there were 10–15 degrees [above zero] I did not go out, I waited for the next day. Not here. I’ve learned enjoying the cold.” (Woman, 39 years)*.

**Outdoors activities ‘Italian Style.’** Despite embracing social norms and habits related to green exercise, some informants perceived challenges and barriers. For example, the weather (especially in the winter) is a recognised barrier that influences the Italians’ participation in and attitudes towards green exercise.

*“We try to spend as much time as possible outdoors, with the limit of my ‘Italianises’ … Sleeping in a tent in winter, I wouldn’t do it. Especially if it’s super cold, like around 15 degrees below zero, you can forget it.” (Woman, 39 years)*.

Moreover, the social norms around the practice of green exercise are so present in Norwegian culture that it may sometimes be perceived as an imposition, eliciting negative evaluations.

*“Cross-country skiing, I was forced to do it. Yes, yes, [I do cross-country skiing] but only when there is nice weather and not if there are 20 degrees below zero. For instance, Sunday I did 20 km, at my own pace, there was nice weather, then yes. But when it’s something you do just because you must, then no.” (Man, 33 years)*.

## Discussions

### Significance of green exercise for health and wellbeing

The logistic regression demonstrated the importance of Green exercise as a factor associated with higher SWL and, to a lesser extent, SRH among the Italian immigrants. The in-depth interviews also support that everyday experience of nature and green exercise is perceived as an important contributor to wellbeing and overall satisfaction, rather than physical health, primarily through relaxation and stress reduction. The explained variance for the association of Green exercise with SRH was relatively low (4%). The inclusion of the control variables led to an increased explained variance, however, the reduced relationship of green exercise with SRH in the controlled model indicates that Educational level and Language proficiency (as indicator of socioeconomic status and acculturation, respectively) have a major impact on people’s SRH. Differently, the explained variance for the association of Green exercise with SWL was higher (10%), showing a substantial contribution to the overall explained variance in the controlled model, as suggested by the relatively small reduction of the association when the control variables were included. Indeed, Green exercise appears to account for almost half of the explained variance for SWL in the controlled model. These findings are partly in keeping with previous research. While some studies found significant associations of green exercise with both SRH and SWL [9, 11], a German study found that participation in leisure outdoor activities was significantly and positively associated with SWL but not SRH [10]. Moreover, review studies of experimental trials found more consistent effects of green exercise on indicators of wellbeing such as self-reported psychological states, as opposed to indicators of physical health such as blood pressure or stress hormones [14, 15]. The findings of the present study indicate that such patterns can be extended to immigrant populations, while at the same time showing how immigration-related factors can influence the salutogenic benefits of green exercise, even among highly skilled and resourceful immigrants from relatively similar cultural contexts, as for the case of the Italians in Norway. Moreover, the findings emphasize the complexity of the pathways linking green exercise with health and wellbeing among immigrants, which will be discussed more in depth in the paragraphs below.

### Appreciation of nature and green exercise

The qualitative analysis highlights that the Italian immigrants generally noticed and appreciated the greater accessibility to nature in Norway compared to Italy. The participants reported to seek contact with nature to reduce stress, but also to enjoy pleasant leisure activities, alone or in company. Accordingly, the descriptive statistics indicate that many Italian immigrants have frequent experiences in nature, most of whom reported to engage in some green exercise during a regular week. Indeed, as described in the introduction, not only the Italian immigrants in Norway practice green exercise in a similar extent compared to the general Norwegian population [25], but they also show considerably larger participation ratings compared to the Italians living in Italy [66], which suggests that the Italian immigrants tend to increase their practice of green exercise after moving to Norway. As emphasized by our qualitative analysis, this seems to be partly facilitated by the greater availability of natural environments in Norway compared to Italy. The perceived wellbeing benefits also seem to contribute supporting the practice of green exercise among the Italian immigrants. The anticipated psychological benefits of being in contact with nature as well as having an emotional attachment to nature, both of which can be strengthened through experiences in nature [13, 67, 68], are important psychological factors supporting the practice of green exercise. For instance, a Norwegian national survey showed that the desire of experiencing nature was a primary motive predicting green exercise behaviour [69]. Qualitative studies from Norway and the U.K. also indicate that green exercise is often motivated by the desire of engaging with natural qualities for physical and cognitive restoration [70], which ascribe meaning and value to health and green exercise [71]. In this respect, it is important to consider the Italians’ cultural understanding of nature. For instance, interviewees mentioned valuing aesthetical elements, such as the sight of a deer, which represents a typical, romantic relationship to nature [72]. This suggests that the Italian immigrants’ notions and definitions of nature may not differ considerably from the hegemonic understanding among the Norwegian population [73].

### Novelty vs. familiarity

In the interviews, it is evident that, despite some recognised differences between Italian and Norwegian nature, the participants were familiar with some characteristics of the Norwegian landscape. Moreover, in keeping with the quantitative measures, many had previously (in Italy) engaged in green exercise, though activities such as hiking and downhill skiing. This may suggest that the familiarity with this activity facilitated the practice of green exercise among immigrants. In this respect, an interesting extension was provided, though the quantitative finings, by the negative association of Childhood experiences with SWL, which indicates that those who had less frequent experiences with green exercise during their childhood were more likely to gain greater wellbeing benefits from practicing green exercise as adults in Norway. Cross-sectional studies have previously demonstrated that childhood experiences with green exercise is a major predictor of green exercise in adulthood [13, 57]. Moreover, previous studies showed that immigrants in Norway who come from countries where green exercise is little practiced, tend to prefer sedentary outdoor activities such as having a picnic or resting [74], rather than practicing ‘active’ forms of green exercise such as hiking in the forest or mountains [75]. Nevertheless, the present study’s findings indicate that not only Italian immigrants who had little experience with exercise during childhood can learn to embrace the green exercise culture in Norway, but this practice tend to be associated with greater wellbeing among the ‘novice practitioners’ compared with those who were more accustomed to this activity.

### Social facilitation

The multivariate logistic regression indicated that greater Social support for green exercise is a highly significant and independent predictor of higher SWL, indicating that the social context plays a key role in the pathways that link green exercise to wellbeing among Italian immigrants in Norway. From the interviews, it is evident that the social component is a heavily present element in the Italian immigrants’ practice of green exercise. This is in line with previous research demonstrating that social support and other social aspects are important facilitators of green exercise [13], which contribute and strengthen the wellbeing effects associated with this activity [4]. Indeed, it has been argued that sociability and social support can be both a factor promoting and supporting the practice of green exercise, as well as a desirable outcome of this practice [1, 4]. The findings are also in line with previous research involving other immigrant groups in Norway. For instance, a qualitative study found that, among immigrant women in Norway, nature experiences and green exercise often included leisure pursuits with close friends or family members [26]. However, an unexpected expansion provided by the qualitative findings suggests that Italian immigrants may, at times, negatively evaluate the social norm (perceived as ‘social pressure’) surrounding the practice of green exercise in Norway.

### Green exercise and acculturation

Previous studies indicate that immigrants may experience barriers to green exercise due to unfamiliarity with using nature for leisure, difficulties in retrieving information on how to reach places where they can hike, swim, ski, etc., but also because of economic barriers [27, 76]. Through a process of acculturation, immigrants may overcome these barriers and integrate new forms of green exercise in their lives. The multivariate logistic regression showed that Language proficiency, as a general indicator of the immigrants’ acculturation, was a highly significant predictor of both SRH and SWL. This suggests that general acculturation is important for immigrants’ health and wellbeing, independently or even beyond the practice of green exercise. That said, the intertwined effect of language proficiency and green exercise on SRH and SWL strengthen the idea that acculturation may take different paths in reinforcing the process of becoming part of a new society. That is, participating in green exercise, which is most relevant in the Norwegian context, may be seen both as the result of acculturation but also as a way of developing a sense of belonging to a new community and promoting the knowledge of the language and other aspects of the Norwegian society. The qualitative findings provide interesting extensions, highlighting how Italian immigrants noticed differences in their cultural practice of green exercise ‘Italian style’ compared to what they interpret as ‘Norwegian.’ In particular, our analysis highlights that positive evaluation of green exercise may be achieved when coupled with adaptation to ones established cultural norms, such as starting to appreciate being out in the cold, but only to a certain degree, or hiking at a comfortable pace.

### Strengths and limitations

This study expands current knowledge on the relationship of green exercise and human-nature interactions with the health and wellbeing of individuals, particularly in the context of intra-EEA migration. Additionally, it provides novel insights into the experiences of Italian immigrants, a group that has received limited research attention. By employing a mixed methods methodology, we were able to establish statistical evidence on the association among green exercise, relevant correlates, and the health and wellbeing of Italian immigrants. Additionally, we gained a comprehensive understanding of their perceptions and experiences through qualitative analysis. The study adhered to rigorous and transparent procedures for conducting mixed-methods research.

However, the study has several limitations. Firstly, it focuses specifically on Italians living in Norway, and the findings may not be applicable to Italian migration patterns in other countries due to potential differences in socio-demographic and migration-related characteristics. It is important to note that Italian immigrants in Norway tend to have higher levels of education, potentially reflecting unique migration trends and the appeal of Norway as a destination for voluntary migrants, but which might have provided a limited overview on the phenomenon.

Another limitation related to the recruitment process for the survey (quantitative strand). It was not possible to access a complete and updated contact list of Italian residents in Norway, which restricted our ability to employ randomized or stratified sampling. The use of the Italian Embassy’s newsletter, COMITES Oslo’s mail-list, and groups on social media to recruit participants may have inflated the proportion of individuals that are affiliated to such groups. We mitigated this limitation by oversampling the dataset to balance key socio-demographic characteristics, thereby enhancing the representativeness of our sample. This was done through the ADASYN approach, which creates artificial examples that closely mirrored respondents’ answers, minimizing distinguishability. However, it is important to acknowledge that such oversampling approaches may inadvertently amplify patterns in the data, potentially overestimating analytical results. Fortunately, the dataset employed in this study does not appear to have been affected by this issue.

While some of the instruments used in this study, such as SRH and SWL, have been previously validated and widely employed in population studies, the validity of other instruments, such as those related to green exercise, childhood experiences, and social support, is less established. These variables were constructed using individual items retrieved from a previous survey conducted by a professional statistics agency (Ipsos MMI). The same dataset has been utilized in a subsequent study with a similar approach [13]. To address the limitations of our approach, we provide detailed descriptions of the instruments, present descriptive statistics for individual items, and conduct preliminary analyses to assess internal consistency. However, it is essential to exercise caution regarding the limited validity of these variables. Limitations in the qualitative part of the study need also to be reported. The use of snowball sampling may have reduced variation in the sample as informants were recruited trough the researchers’ network. This sampling strategy limits the generalizability of the qualitative findings, and effort to include large variation in socio-demographic characteristics and experiences of being and Italian in Norway should be made in future research.

## Conclusions

The findings of the present mixed-method investigation indicate that Italian immigrants to Norway appreciate the greater opportunities for green exercise in Norway compared with Italy, and experience this as beneficial to their health and wellbeing. The study sheds light on the complex pathways that link green exercise with health and wellbeing, highlighting the specific challenges and barriers experienced by this group of immigrants in relation to the practice of green exercise. The study also emphasizes the importance of framing this phenomenon under the prism of acculturation as, in the Norwegian context, *friluftsliv* is a specific culturally informed practice of being healthy and can be seen as an important aspect in becoming part of a new society. Although this study focuses specifically on Italian immigrants to Norway, the findings provide valuable knowledge for understanding green exercise participation among other immigrant groups. Moreover, this study highlights that more research is needed to understand how different immigrant groups, including those from EEA countries, practice green exercise in their own acculturated ways.

## Data Availability

The datasets supporting the conclusions of this article are available from the corresponding author on reasonable request.

## List of Abbreviations

EEA: European Economic Area
CI: Confidence Interval
OR: Odds Ratio
SRH: Self-rated health
SWL: Satisfaction with life
W: Wald Statistic
